# Experiences and outcomes of craft skill learning with a 360° virtual learning environment and a head-mounted display

**DOI:** 10.1016/j.heliyon.2020.e04705

**Published:** 2020-08-21

**Authors:** Susanne Hallberg, Laura Hirsto, Jani Kaasinen

**Affiliations:** a University of Eastern Finland, Finland; b University of Eastern Finland, University of Helsinki, Yliopistokatu 2, 80100 Joensuu, Finland

**Keywords:** Improving classroom teaching, Pedagogical issues, 360° VLE application in craft learning, Teaching and learning strategies, Multimedia/hypermedia systems, Mobile learning, Education, Educational psychology, Evidence-based education, Pedagogy, Teaching research, Psychology

## Abstract

Virtual reality environments (VLEs) such as 360° videos have been introduced as educational tools over the last few years, although the pedagogical value of these media has not been widely examined, especially in the context of craft skill learning. Moreover, emotions and competences have a great impact on the usability and adoption of ICT – and on learning. In this paper, mixed-method strategies were used to address these pedagogical and emotional needs in the context of craft learning and 360° VLE. Furthermore, a quasi-experimental design was used to compare learning outcomes of 360° VLE and traditional groups. Findings based on quantitative analysis suggest that negative or positive ICT-attitude did not affect how students experienced traditional or 360° lessons emotionally. However, ICT- and craft-competences had significant correlations with the described emotions. No significant differences in terms of learning outcomes were observed between the traditional and 360° teaching methods. According to the thematic analysis of the interviews, the 1^st^-person-view 360° VLE could be used for basic skill observation and visualization to support traditional hands-on learning. Moreover, a head-mounted display was considered to help with focusing on the demonstration. However, more interaction with the interface and opportunities for direct interaction with the instructor were seen as necessary in 360° VLEs for skill learning in the future.

## 1 Introduction

Learning a craft skill is a complex process, which includes know-how about working postures, materials and tools (Tynjälä, 2007). This knowledge is procedural and tacit by nature (Bereiter, 2002; Toom, 2012), and becomes visible through actions (e.g. Syrjäläinen, 2003). According to Dewey (1916), this knowledge is socially constructed, and hence blended in communities of practice and transmitted by interaction, e.g. between a master and the apprentice, who observes the actions of the expert. Further, transmission of this knowledge, i.e. skill learning, requires a “learning-by-doing” symbiosis between learner and the objects, such as materials and tools (Dewey, 1916). In this way, a motor skill develops gradually by practicing. Nevertheless, the instructor's role in motor skill learning is to provide a model, scaffolding and feedback for the learner (Moore, 1989). Moreover, by verbalizing his/her actions the instructor offers a model of the used procedures and metacognitions for the learner (Syrjäläinen, 2003).

However, it can sometimes be difficult to access this know-how, due to distance and the contemporary and silent nature of procedural knowledge. To overcome this problem, various multimedia technologies, such as Youtube-videos, have widely been used to mediate and visualize gestural know-how (e.g. Kim, 2011; Glushkova and Manitsaris, 2018). Currently, 360° videos and head-mounted displays (HMD) are accessible in education, due to decreasing costs and wider availability of contents (Zhou et al., 2018). Furthermore, 360° videos are the most effortless way to create immersive VR-content in HMDs (Tham et al., 2018).

Nevertheless, there is a lack of studies examining the pedagogical value of 360° media and HMD use. However, when adopting new technology for education, three fundamental stages of learning - conceptualization, construction and dialogue, - should be considered (Mayes and Fowler, 1999; Fowler, 2015). In this paper, the term e-learning is used to refer widely to the educational use of technical devices. The definition includes ICT (i.e. information and communication technology) mediated instructional material and pedagogical methods that are used to promote learning (Clark and Mayer, 2008; Juutinen, 2011).

Characteristics of learning environments or ICT -tools *themselves* do not directly promote learning, but they can afford specific learning tasks which potentially benefit learning (Bower, 2008; Dalgarno and Lee, 2010). These potential characteristics can be defined as *educational affordances* (Norman, 1988). Since ICT develops constantly, it is more reasonable to observe affordances instead of specific technological properties of ICT in an educational context (Bower and Sturman, 2015). Further, different learning tasks have different affordance requirements which the ICT-tool in use should fulfill (e.g. Bower, 2008). Thus, it is crucial to examine educational affordances of ICT-tools in various contexts to facilitate functional and high-quality learning situations.

Moreover, the quality of e-learning is firmly determined by the satisfaction of the students (Soffer and Nachmias, 2018), including emotional experiences (e.g. Juutinen, 2011). Hence, in this study we focus on the potential affordances that HMD and 360° virtual learning environment (VLE) offer in the skill learning process, keeping the elements of learner satisfaction in mind.

### 1.1 Emotions, attitudes and competence in e-learning

E-learner satisfaction is dependent on learners' attitudes and emotions toward ICT, self-efficacy, continuous interaction with the instructor and the instructor's attitude, flexibility and quality of the course and of the technology, perceived ease of use and usefulness of the technology, diversity in assessment and finally, interaction with others (Sun et al., 2008). In this study, the prior interest lies in emotions and interaction, since they are important elements of successful learning both in traditional (e.g. Järvelä, 2011) and e-learning contexts (Picciano, 2002; Wanstreet, 2006; Sun et al., 2008; So and Brush, 2008; Juutinen, 2011; Soffer and Nachmias, 2018). Furthermore, emotions have a great impact on the quality of human-technology interaction (Saariluoma and Jokinen, 2014), and the significance of this interaction is emphasized in e-learning due to the possible separation of the learner and the instructor (Soffer and Nachmias, 2018).

Furthermore, earlier experiences, emotions and attitudes towards technology may affect how one perceives novel technology (Juutinen, 2011; Saariluoma and Jokinen, 2014). For example, according to Juutinen (2011), successful ICT-experiences foster positive emotions leading to a cycle of pride and competence, whereas frequent negative experiences and emotions may lead to a cycle of frustration. This also affects education, since positive perceptions concerning a system and its perceived benefit for learning, i.e. usefulness, are associated with satisfaction (Sun et al., 2008) and more effective and efficient learning (Sun, 2015). Thus, the emotional usability of ICT impacts on whether a learner is willing to use a device or application at all (Juutinen, 2011).

*Competence* is used in this paper to describe the emotional user experience, which includes efficacy and emotions (Gravill et al., 2006; Saariluoma and Jokinen, 2014). *Self-efficacy* means an individual's belief or evaluation of success concerning a certain task (Bandura, 1982, 2018; Marakas et al., 1998; Sun et al., 2008), and has for example been associated with better learning outcomes and higher student enrolment in a web-based course (Further, Wang & Newlin, 2002). A learner's positive perception of his/her competence further improves satisfaction (Saariluoma and Jokinen, 2014).

### 1.2 Virtual learning environment and immersion

In their review, Dalgarno and Lee (2010) have identified potential learning affordances in high-end 3D-virtual learning environments (VLEs): VLEs can be utilized to facilitate learning tasks that lead to spatial knowledge representation, engagement and contextual, experimental and collaborative learning. These characteristics can be also found to some extent in the combination of 360°-video and head mounted display (HMD).

The feature that separates virtual reality technologies from other media is the ability to create *immersive* virtual environments through interactivity and sensory feedback. The intensity of physical immersion depends on the media and a device, aka a virtual reality system, and user behavior, such as involvement (Wirth et al., 2007). The term *VR* (virtual reality) is usually used with advanced technology which provides physical or sensory immersion through various senses and enables movement and object manipulation, leading to mental immersion and a strong feeling of being engaged and present in a virtual environment (Sherman and Craig, 2003).

Further, the place illusion appears to enhance engagement and thus to direct the viewer's focus to the learning content (Rupp et al., 2019). Consequently, less mind wandering in an online learning context correlates with better academic results (Hollis and Was, 2016), and reduced distractions in a virtual environment may lead to improved conceptual and spatial learning (Tüzün and Özdinç, 2016). Moreover, HMD isolates the user's senses from the physical world, which eliminates certain environmental distractions, such as noise and visual disruptions (Higuera-Trujillo et al., 2017) and thus may aid concentration.

Furthermore, immersion or feeling present are related to increased interest in the subject matter and greater positive affect (Rupp et al., 2019). Similarly, in their review of HMDs and vr-systems used in education and training, Jensen and Konradsen (2018) found that a strong feeling of immersion generally has a positive effect on learning outcomes, even though one study pointed out that a too immersive experience may also confuse and distract students from the task. Overall, according to Fowler (2015), immersion provides a concept that can link technological, psychological and pedagogical aspects of learning in virtual environments.

### 1.3 Learning outcomes and satisfaction: 360° VLE versus traditional

There is a lack of studies comparing the use of 360° VLE and HMD technology to traditional teaching according to learner satisfaction and learning results, especially in the skill-learning context. Hence, relative studies are examined in this paper. Consequently, according to Dixson (2010), online instruction and traditional instruction can be equally effective, if instructor presence and active/cooperative learning are provided. For example, equal learning outcomes have been reported between academic online and face-to-face courses (Soffer and Nachmias, 2018) and between a traditional classroom lesson and an augmented-reality based learning method (Furió et al., 2015).

Concerning student satisfaction, there have not been statistically significant differences between traditional and distance learning environments (Allen et al., 2002). However, Soffer & Nachmias (2018) reported higher measures in engagement and satisfaction in online courses in comparison to the face-to-face format. Similarly, when comparing 360° video to other media, Harrington, Kavanagh, Wright Ballester, G., Wright Ballester, A., Dicker, Traynor, et al. (2018) found higher engagement in students studying with 360° video than in students watching 2D video. Furthermore, Virtanen et al. (2017) reported that medical students were mostly satisfied with a 360° virtual laboratory in comparison to a web-based environment, but needed clearer instructions, technical support and more supervision from the teacher. Similarly, So and Brush (2008) encouraged the provision of students with social interaction in distance learning environments.

### 1.4 Skill conceptualization - observational learning with 360° VLE

In the craft learning context, visuomotor skills and hand-eye coordination are essential, with diverse roles in the different stages of skill learning. When beginning to learn a new skill, a learner first forms an initial understanding of the objective of learning (Fowler, 2015), and the visual cues are emphasized. According to Fowler (2015), this *conceptualization* can be supported by instructions and demonstrations, either traditionally face-to-face or with multimedia representations. In this research, HMD was used to view the instructional 360° video. 360° video is omnidirectional and panoramic, i.e. it enables viewers to change the view in an unbroken circle (c.f. Rupp et al., 2019).

The combination of a HMD and a 360° video may offer unique opportunities for observation due to a 1^st^-person view and the possibility to explore learning space naturally by head movements. Although the VLE in this research is semi-immersive due to limited possibilities for environmental interaction (Fowler, 2015), it may afford more feeling of involvement and interactivity due to head movement in comparison with an ordinary video which is watched from a fixed point (Harrington et al., 2018). Moreover, presence-related psychological responses (Higuera-Trujillo et al., 2017) and increased subject matter interest (Rupp et al., 2019) have been found even with less immersive smartphone applications and HMDs.

With a 1^st^-person view, a viewer sees the experts’ body and hands instead of his/her own when s/he looks toward him/herself. Thus, the viewer may feel the virtual body as if it his/her own and create cognitive maps from the sensory cues of the video (Slater and Sanchez-Vives, 2016). Studies concerning neurorehabilitation show that intentional action observation and motor imagining activate the same brain regions that are stimulated in actual movement, thus enhancing motor learning (Adamovich et al., 2009; Eaves et al., 2016). Furthermore, this may evoke physiological responses similar to those associated with being in physical space (Higuera-Trujillo et al., 2017).

However, since the viewer has a possibility to direct his or her attention anywhere in the 360° image, visual and audio guidance towards the desired spot, for example skill demonstration, is considered to be beneficial (Sarker, 2017). This is especially important when a 360° video follows a directed narrative (Sherman and Craig, 2003; Wirth et al., 2007). Well-designed guidance may lead to a positive experience; on the other hand, if the viewer does not know where to look, s/he might end up looking in the wrong direction, leading to a negative experience (Sarker, 2017). However, if the viewer is highly interested in the content, s/he may voluntarily direct attention towards it (Wirth et al., 2007).

### 1.5 Skill construction - interaction with the instructor, materials, tools and interface

After a learner has formed an initial conception of the skill, s/he explores the idea in practice to examine how actions impact in reality and to gain tactile feedback. Hence, this phase of *construction* requires interactivity (Fowler, 2015). Furthermore, in the context of traditional craft-learning and e-learning, three types of interaction should be considered: interaction with a) materials and tools, b) the instructor and c) content (Moore, 1989) and interface (Hillman et al., 1994).

First, when practicing carving, a learner constantly receives feedback through different senses about the technique, materials and tools (Suojanen, 1993). For example, when a knife is squeezed in the hand with excessive force, the knuckles turn white, the knife bites too big chunks of wood and even certain types of noises can be heard. According to Moore (1989), this construction phase is the time when interaction with the instructor becomes most valuable, since s/he can provide feedback for the learner about correct application and intensity or the extent of desirable activity. For example, the instructor may decode those different sensations of the learner and help him/her forward: perhaps the knife is blunt, or the technique should be improved. Furthermore, the instructor provides scaffolding, answers questions and encourages discussion (Moore, 1989; Dixson, 2010). In a skill learning context, this is a very traditional way of learning, e.g. master and apprentice, due to procedural, situated and silent kind knowledge which becomes available through dialogue (Fowler, 2015).

However, in the case of remote learning, e-learning environments have been criticized since they tend to lack human interaction and communication (Moore, 1989; So and Brush, 2008). Concerning interaction, communication with instructor and peers seems to correlate with higher engagement (Soffer and Nachmias, 2018; Dixson, 2010), satisfaction and perceived learning (Soffer and Nachmias, 2018; Sher, 2009; Bulu, 2012), as well as with motivation and learning outcomes (Soffer and Nachmias, 2018; Du et al., 2005). Timely feedback from an instructor has a great impact on a student's perceived satisfaction in e-learning (Sun et al., 2008; Virtanen et al., 2017; Arbaugh, 2018), as does the instructor's attitude (Sun et al., 2008). Further, the instructor's presence is considered important for effective e-learning, and thus it is important to provide various channels of communication for students (Dixson, 2010).

Concerning the interaction with content and interface in the e-learning context, the interactivity of video instructions enhances learning and satisfaction (Zhang et al., 2006). Consequently, in VLE it is beneficial that the learner has opportunities for self-produced movements with simultaneous visual feedback (Held and Hein, 1963; Slater and Sanchez-Vives, 2016). Moreover, possibilities to control and manipulate content in virtual reality appear to invoke feelings of autonomy, motivation and engagement (Tham et al., 2018). Furthermore, the virtual model of a skill should correspond to real-world tasks in order to be transferred correctly to practice (Slater and Sanchez-Vives, 2016) and similarly, simulated body movements should correspond to the physical body, since a great feeling of impairment may cause nausea, thus disturbing the learning experience (Lackner, 2014). With current tracking technology, body movements can be precisely simulated, but haptic correspondent which is necessary to craft skill learning is hard to fulfill (Slater and Sanchez-Vives, 2016; Jensen and Konradsen, 2018). Nonetheless, the need and quality of interactivity in an e-learning context depends on the skill and its degree of difficulty. For example, Nousiainen et al. (2008) found that in the learning of basic surgical knot tying skills, an ordinary video was as effective a medium as an interactive one.

### 1.6 Motivation and objectives of the current study

The aim of this paper was to examine university students’ perceived satisfaction of a first-person perspective 360° virtual learning environment (VLE) and a head-mounted display (HMD) in a handcraft skill learning process and the learning outcomes of a specific craft skill. Concerning satisfaction, we were mainly interested in attitudes, competence and emotional experiences of ICT-use, since they can affect learning and predict the adoption and use of ICT-devices. Moreover, we found no earlier studies concerning the utilization of HMDs and 360° VLEs in a craft learning context. Thus, we created the following research questions (RQs):

RQ1. How are the students’ ICT attitude, ICT competence and craft competence related to emotions that experienced after the craft lesson?

RQ2. How do the learning outcomes differ between the attendees of 360° VLE and traditional craft lessons?

RQ3. What is the perceived level of student satisfaction with a HMD and a 360° VLE in craft learning?

## 2 Material and methods

### 2.1 Participants

During the spring of 2018 a group of kindergarten teacher students (n = 16) in a Finnish university participated in a crafts course as part of their compulsory studies. The aim of the course was to practice basic craft skills and learn how to teach these skills to children. As part of the course, students taught craft skills to the rest of the group, e.g. sawing and metal bending but excluding carving, which was taught by the first author. The students were 21–53 years old and the majority were female (15 out of 16), which is customary in Finnish early education teacher studies. Therefore, it was not reasonable to study the impact of gender in this study. All the participants gave their informed consent to participate in the study. In Finland, it is not required to have preliminary ethical approvement of this kind of design.

### 2.2 Context of the study

**Figure 1:**
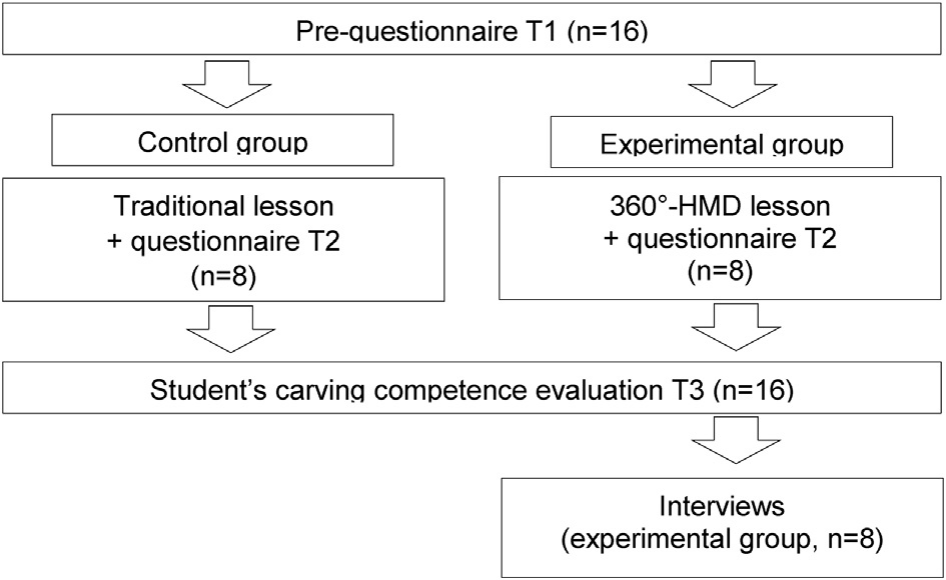
The experimental design of the study.

A quasi-experimental design was used (Figure 1), and there were two groups for comparison. For the experiment, we manipulated the learning method to explore its impact on learning outcomes. Hence, the students of the control group attended a traditional crafts class, whereas the experimental group attended a lesson which was given via a HMD and a 360° video.

**Figure 2:**
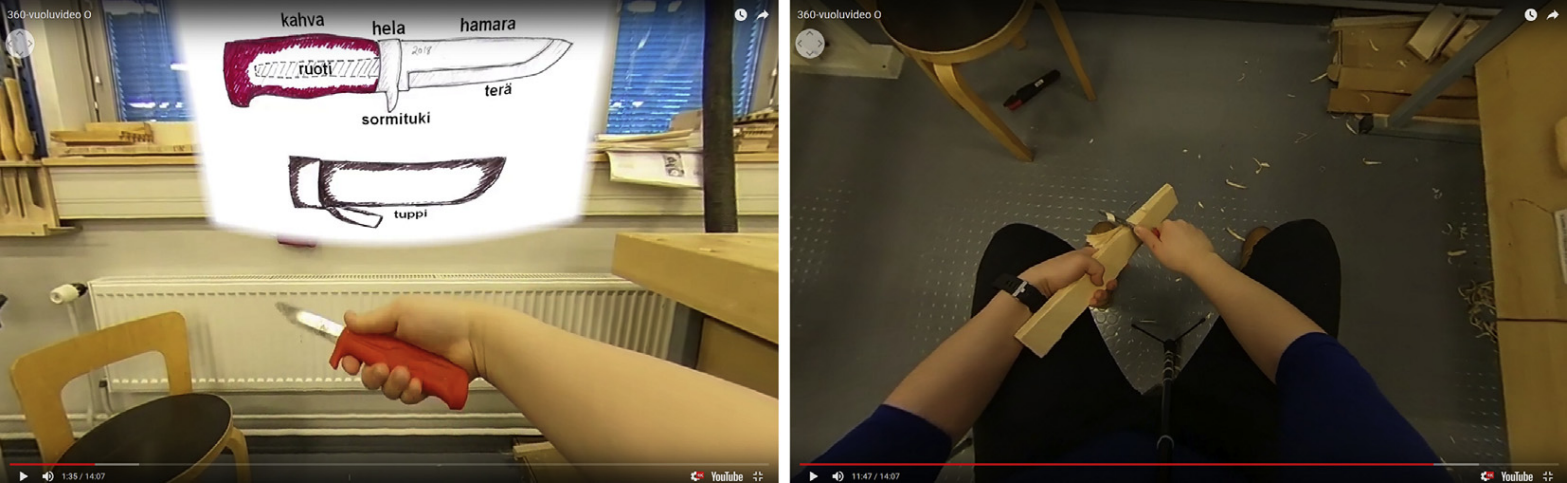
Screenshots from the video.

For the experimental design, the first author designed and created the 360° craft learning video (see Figure 2). The educational goals of the video were determined as: (1) to understand how to teach carving to kindergarten children safely, (2) to understand some theoretical elements of carving and (3) to learn correct carving techniques. We were mainly interested in goal number three, the acquisition of the technique. The video included visual demonstration and verbal explanation about the correct carving technique, working posture, safety and theory of carving.

The HMD used in this research was a handheld View-Master Virtual Reality viewer with a Samsung Galaxy S6/S6 edge smartphone and basic on-ear headphones. Video was watched using the Youtube and Cardboard Android apps. Head tracking and a lever for clicking the interface were enabled. The video was filmed with a Ricoch Theta V 360° camera and edited with Adobe Premiere Pro CC.

Mixed methods were used to collect data in order to gain a broader understanding of the research questions: quantitative methods are well suited to experimental design, whereas qualitative methods help to gain a deeper understanding of one's subjective experiences (Creswell and Plano Clark, 2018), which was our main interest in this study. Thus, the instruments for collecting data included an experimental scenario, pre- and post-test questionnaires, an observation form for the carving competence evaluation and interviews for the experimental group.

First, all participants (N = 16) completed the T1-questionnaire regarding their ICT- and craft competences and attitudes towards ICT-usage in their studies. Participants were assigned into a traditional group and a 360° VLE group based on their answers; the aim was to create two similar groups. Possible health issues concerning HMD were also asked, although no detailed description of the issue was required. Thus, if a participant for instance reported having a risk of simulator sickness, s/he was assigned to the traditional group. Furthermore, those who did not want to be interviewed were also assigned to the traditional group since we were mainly interested in the experiences arising from the 360° VLE class.

After the group formation, the lessons were conducted in the university. The control group participated in a traditional class as a group (n = 8) in a wood workshop during the scheduled craft course. The participants of the experimental group (n = 8), however, attended the 360° VLE lesson individually during a one-week period. Each of them was allowed to make an appointment for the lesson, since the 360° VLE -experiments had to be conducted amidst their regular studies and other courses.

The experiment was conducted in a small office room and the participant viewed the video while seated. Usage of the head-mounted display was introduced: how to explore the view by head movements and how to pause and play the video. The duration of the video was approx. 15 minutes, and the first author was present in the room during the test. The T2-questionnaire was conducted immediately after the lessons in both cases. Evaluation of the content of the craft lessons was also investigated after the lessons (T2), and there were no statistically significant differences between the groups (Table 1). Thus, it can be concluded that the possible differences between groups did not result from the differences in the content of 360° video and traditional lessons.

**Table 1:** Reliability of sum-items according to Cronbach's alpha (α).

Sum-item	Items (n)	α	An example item
**T1 – questionnaire**			

Negative ICT-attitude	8	.900	Q20. ICT-usage in studies… makes me anxious
Positive ICT-attitude	5	.911	Q6. When a new ICT-device for studies is introduced, I am excited
ICT-competence	5	.882	Q5. Learning to use new ICT-devices is usually very easy for me
Craft-competence	4	.862	Q29.How would you evaluate your current carving skill? Q9. I didn't learn anything new from the carving lesson∗

**T2 – questionnaire**			

Negative emotions	4	.735	Q21/Q2 During the class, I felt…frustration
Positive emotions	4	.632	Q25/Q6. During the class, I felt…excitement
Evaluation score of the lesson content	5	.676	Q2. Techniques were demonstrated remarkably well
Self-evaluated learning	4	.815	Q15. Based on this class…I believe I will be able to choose a suitable and secure carving knife for children's use.

**T3 – skill assessment scale**			

Carving technique	4	.683	Q13. Use of force is controlled (no white knuckles)
Working posture	6	.778	Q7. The elbow (holding the wood block) is supported by the knee.
Safety	5	∗∗	Q14. The blade is directed away from the worker

∗ This question was included in the T2-form.

∗∗ α could not be calculated, because nearly all the results concerning safety were identical.

After the traditional group lesson and all the individual 360° VLE -lessons were carried out, the students’ carving competences were assessed by evaluating their carving skills. All the carving skill evaluations were conducted during the same day and there was approximately a one-week gap between the lesson and the evaluation. It should be noted that all students were instructed to not try carving themselves before the evaluation. Every student performed carving for a couple of minutes and their performance was evaluated by the first author. There were no other people in the room at the same time.

### 2.3 Measurement items and interview questions

No existing instrument was found which would adequately address the research questions, and therefore two questionnaires and nine sum-items were created for measuring the students' ICT attitude, ICT competence, craft competence and emotions after craft lessons. The pre-test T1 aimed to evaluate the students' prior competences and attitudes concerning ICT and crafts, since these could have an influence on emotions after the craft class. Sum-items for measuring ICT attitude (negative and positive) were adopted from the “emotional effects of e-learning in Finnish government office”*-* questionnaire developed by Juutinen (2011). The sum item for ICT competence was a combination of Juutinen's (2011) questionnaire and ICT competence measures from the TPACK-questionnaire (Valtonen et al., 2017).

The sum-items for negative and positive emotions after craft lessons (T2) were slightly modified from ”emotional questionnaire items” by Saariluoma and Jokinen (2014). Finally, items concerning crafts, including craft competence (T1), students’ evaluation of the craft class content (T2) and evaluation criteria for learning outcomes (T3), were developed by the first author and the third author in consideration of the pedagogy of craft learning. In order to keep the questionnaires compact enough for students to complete it during their studies, all the adopted questionnaires were compressed. A six-point Likert scale was used in all items ranging from 1 (strongly disagree) to 6 (strongly agree), with the exception of the T3-form for carving competence observation, in which the evaluation of independent items was a dichotomy. In T3, maximum points for sum-items are equal to the items they include.

In this study Cronbach's alpha (α) was used to assess the reliability of the sum-items, as seen in Table 1. According to Metsämuuronen (2011), the value of alfa must be over 0.60 for acceptable reliability of the sum item. Further, survey data were analyzed through descriptive statistics and non-parametric tests, since the sample size was small (N = 16, <30) and variables were ordinally scaled (Metsämuuronen, 2011). Kendall tau-b was chosen to examine correlations between variables, since it is more accurate with small samples in comparison to Spearman. One goal of the analysis was to identify whether there were any distinguishable differences between the experimental group and the control group according to emotions and learning outcomes. Furthermore, the aim was to observe how these emotions might be related to craft competence, ICT competence and ICT attitudes overall. In order to create comparable groups for testing emotions, K-means clusters were used. Moreover, a Mann-Whitney U-test was used to compare two independent groups in non-parametric settings.

Further feedback from the 360° VLE and the HMD method was collected from the participants of the experimental group (N = 8) individually via semi-structured theme interviews. The semi-structured theme interview is often used when the participants share a similar experience, in this case the 360° VLE carving lesson. Interview themes are included in Appendix 1. The interviews were recorded as audio files for transcription and analysis, and the total duration of the interviews was approximately 71 min. Examples of the interview questions include: “Describe your expectations and thoughts, as you arrived at the 360° VLE lesson”, “What did you like/dislike about the learning method?”, “What would you think if the whole craft course would be taught via a 360° VLE and a HMD?” and “Describe what an optimal 360° VLE lesson would include.”

After transcription, each transcript was reviewed individually by the first author in order to identify major themes. The data was subjected to qualitative *theory-guided content analysis* (Sarajärvi and Tuomi, 2018) with process coding and pattern coding methods (Saldaña, 2013). Theory-guided content analysis combines data-driven and theory-driven analysis methods (Sarajärvi and Tuomi, 2018). In current study the analysis began by coding and clustering similar topics deductively, in the data-driven manner. These clusters and themes, in turn, were named after theoretical concepts associated craft skill learning and use of 360° media and head-mounted displays.

Cross validation was conducted with an intercoder reliability procedure (e.g. Cho, 2008) by picking 50 quotes from the data. Another coder (the second author) assigned this data to categories based on the preliminary descriptions. Of the quotes, 82% were placed in matching categories. Clarifications were negotiated until full agreement was reached. In the discussions, descriptions of the categories were defined more clearly and one quote was replaced to another category. The qualitative analysis of the interviews aimed to identify in what ways factors highlighted in the skill learning theory framework were featured in the students’ comments. Thus, a description of the usability of 360° VLEs and HMDs in skill learning was created.

### 2.4 Comparison of the test and control groups

According to the T1-questionnaire, the background variables were tested to form a control group and a test group. Scores from each of the measures were compared using the Mann-Whitney U-test. As seen in Table 2, there were no statistically significant differences between the 360° VLE and the traditional groups regarding craft competence, ICT competence, ICT attitude or age. Thus, the division of students into the test and control croup can be considered equal and representative.

**Table 2:** Mann-Whitney U-test comparing background variables of the 360° VLE (n = 8) and traditional groups (n = 8).

Background variables	U	W	Z	*p*
Positive ICT-attitude	45.5	81.5	1.428	0.161
Negative ICT-attitude	18.5	54.5	-1.420	0.161
Craft-competence	29.0	65.0	-0.316	0.798
ICT-competence	36.5	72.5	0.993	0.336
Age	26.0	62.0	-0.635	0.574
Evaluation score of the lesson content∗	28.5	64.5	-0.381	0.721

∗ The item was included in the T2-questionnaire, after the lessons.

## 3 Results

### 3.1 Profiling students according to positive and negative emotions

In order to examine the emotional aspects of the study, groups of participants were formed using a K-means cluster analysis. Sum items of positive and negative emotions after attending the craft class were included in the analysis. No significant differences were observed in terms of negative emotions. However, there was one outlier observation with highly negative emotions belonging to the control group. This observation had a large distance (Md = 1.59) and was therefore removed from the subsequent analysis regarding emotions. (Positive emotions: n = 1, mean rank = 1.5; Negative emotions: mean rank = 16).

Hence, according to the results two groups of differently profiled students were identified. Cluster 1 had higher positive and lower negative emotions and was named the “highly positive” group. The second group was named the “neutral” group, since it had lower positive emotions and somewhat higher negative emotions compared to the first cluster (Table 3). As also seen in Table 4, the results of the Mann-Whitney U-test showed that there were significant differences in the amount of positive emotions between the clustered groups (p < 0.01). Furthermore, the value of η^2^ was 0.70, indicating an intermediate effect (Cohen, 1988).

**Table 3:** K-means clusters in terms of positive and negative emotions.

Sum-item	Final cluster centers (N = 15)		ANOVA	
	Cluster			
	1 “highly positive” (N = 7)	2 “neutral” (N = 8)	F	*p*
Positive emotions (T2)	4.54	3.63	34.155	.000∗∗∗
Negative emotions (T2)	1.39	1.44	.061	n.s.

∗∗∗*p* < .001.

**Table 4:** A Mann-Whitney U-test on positive and negative emotions by emotion-clusters (N = 15).

Sum-item	Cluster	N	Mean rank	U	*p*	η^2^
Positive emotions (T2)	1	7	12.00	.00	.000∗∗∗	0.7
	2	8	4.50			
Negative emotions (T2)	1	7	8.07	27.5	n.s.	n.s.
	2	8	7.94			

∗∗∗*p* < .001.

### 3.2 RQ1: how are the students’ ICT attitude, ICT competence and craft competence related to emotions experienced after the craft lesson?

The Mann-Whitney U-test was performed to examine whether there existed a significant difference between the “highly positive” and “neutral” emotion groups in terms of ICT attitudes and ICT competence. Regarding the ICT-attitude, the results show (see Table 5) that there was no statistically significant difference between the clustered emotion groups (T2) and positive or negative attitudes (T1) towards ICT. This implies that the student's ICT attitude was not related to the emotions s/he experienced after the craft lesson.

**Table 5:** Mann-Whitney U-test on ICT competence, craft competence and ICT attitudes between the emotion cluster groups.

Sum-item	Cluster	N	Median	Mean Rank	U	*p*	η^2^
ICT-competence	1^a^	7	4.20	10.50	10.5	.04∗	.27
	2^b^	8	3.30	5.81			
Craft-competence	1	7	4.25	11.36	4.5	.004∗∗	.49
	2	8	3.13	5.06			
Positive ICT-attitude	1	7	4.00	9.50	17.5	n.s.	n.s.
	2	8	3.30	6.69			
Negative ICT-attitude	1	7	2.25	7.36	32.5	n.s.	n.s.
	2	8	2.19	8.56			

∗*p* < .05; ∗∗*p* < .01.

a ”highly positive” emotions.

b ”neutral” emotions.

Concerning ICT competence, the U-test results indicated that students who experienced ”highly positive” emotions had stronger ICT competence (Mean rank = 10.5) compared to the ”neutral” group, of which the ICT competence was lower (Mean rank = 5.81). Furthermore, the group with ”highly positive” emotions had significantly (p = 0.004) greater craft competence (Mean Rank = 11.36) than the ”neutral” emotions group (Mean Rank = 5.06), as seen in Table 5.

We were also interested to determine whether there were any emotional differences between the 360° VLE group and the traditional group. As the Mann-Whitney U-test results show in Table 6, there were no statistically significant differences between the traditional and 360° VLE groups in terms of positive and negative emotions after the craft class (T2). Furthermore, the result was non-significant even when the highly negative outlier observation from the traditional group was included in the analysis. Thus, the conclusion is that the traditional and the 360° VLE lessons were equal from the emotional point of view in this case.

**Table 6:** Mann-Whitney U-test on positive and negative emotions between the 360° and traditional groups.

Sum-item	Group		N	Median	Mean Rank	U	p	η^2^
Positive emotions (T2)	1.	Traditional	7	4.5	9.57	17.00	0.232	n.s.
	2.	360°	8	4	6.62			
Negative emotions (T2)	1.		7	1.2	6.93	35.50	0.397	n.s.
	2.		8	1.5	8.94			

### 3.3 RQ2: how do the learning outcomes differ between the attendees of the 360° VLE and traditional craft lessons?

The Mann-Whitney U-test was performed to examine possible differences in carving skills between 360° VLE and traditional groups (Table 7). There was no statistical significance between the traditional group (n = 8) and the 360° VLE group (n = 8) in terms of their learning outcomes, although the 360° VLE group had a high variance concerning the technique item (σ^2^ = 2). Accordingly, this indicates that the 360° VLE and traditional methods were equally effective concerning the learning of this specific skill.

**Table 7:** Mann-Whitney U-test on skill assessment sum items between the 360° VLE and traditional groups.

Sum-item	Group	N	Median	Mean Rank	U	*p*	η^2^
Technique (max. 4 points)	1^a^	8	3.50	10.00	20.00	.234	n.s.
	2^b^	8	2.50	7.00			
Posture (max. 6 p.)	1	8	6.00	10.00	20.00	.234	n.s.
	2	8	4.50	7.00			
Safety (max. 5 p.)	1	8	4.60	8.00	36.00	.721	n.s.
	2	8	4.00	9.00			
All three combined	1	8	4.67	10.62	15.00	.083	n.s.
	2	8	4.00	6.38			

a Traditional group.

b 360° VLE group.

### 3.4 RQ3: what is the perceived level of student satisfaction with a HMD and a 360° VLE in craft learning?

This section presents the results of theory-guided content analysis regarding interviews of the experimental group (N = 8). As can be seen in Table 8, four themes concerning satisfactory craft learning with 360° VLE emerged from the participants’ learning experiences. These themes and their sub-themes are examined next more closely.

**Table 8:** Overview of the themes emerging from the interview data.

Themes	Sub-theme	Category		Codes/quotations/respondents
**Skill conceptualization – observational learning**	Observational learning	Observing from the 1^st^-person perspective		7/8/(4)
		Observing from the 3^rd^-person perspective		4/6 (4)
	Perception/Attention	Concentration-aiding features in 360° VLE		8/13/(5)
		Concentration-disturbing features in 360° VLE		3/6/(3)
		Other concentration-related features		7/7/(5)
**Skill construction – interaction with materials, tools and interface**	Learning and practicing in an authentic environment (Situational learning, specific learning context)	Learning and practicing by doing		7/12/(5)
		Acquiring the conception of the attributes of materials and tools		3/3 (2)
	Learning and practicing in a simulated environment	Interacting in a simulated environment		7/10 (4)
		Moving in a simulated environment		4/5 (2)
		Immersion	Feeling of agency	4/5 (3)
			Feeling of “being there”	6/6 (5)
			Immersion, other	2/2 (2)
**Social interaction in skill learning**	Scaffolding	“ad hoc” scaffoldig	Asking instructions	4/4 (3)
			Teacher as instructor and support	5/5 (4)
		Planned scaffolding	Teacher as a model: demonstration of the skill	3/4 (4)
	Social interaction, other			3/3 (3)
**Perceived learning outcomes**	Past experiences guide the task			6/6 (4)
	Imitating the instructor/video			3/6 (5)
	Visualizing the task			2/2 (2)
	Recalling and remembering the lesson			4/4 (3)

#### 3.4.1 Skill conceptualization – observational learning

The first-person perspective was an essential element of the 360° video. Two participants reported that the working posture became more concrete due to the perspective. One of these two elaborated that understanding the handling of the tool was easier and more natural after watching it from the first-person angle. Similarly, one participant emphasized that imitation of the correct hand postures became more concrete. Two participants also reported that the video helped to clarify and imagine how the activity would look if they worked by themselves. Moreover, concerning the carving situation after the 360° VLE lesson, one participant said that it felt as if s/he would have been there crafting all over again.

Half of the participants mentioned that traditionally there is more distance between the student and the teacher. In other words, teaching or demonstration occurs “somewhere far away”, as one participant expressed. Concerning this, s/he speculated whether s/he would have paid attention to postures in the same way via a third-person perspective. Another participant said:

*It [1*^*st*^*-person perspective] was good, because I learn by “someone guiding my hand”… You saw what was happening all along. If it [the video] had been filmed differently, like I would've watched you demonstrating over there, I wouldn't have learned the same way, because I wouldn't have seen with my own eyes what was happening. (H8)*

One participant approached the topic from the viewpoint of a teacher, and consequently the 360° video could help the teacher to demonstrate certain skills:

*Demonstration looks easy that way [via 360° video.] It seemed quite functional. For example, if you think about knitting, it is challenging to begin to demonstrate it next to someone, in comparison to showing it as if you're doing it yourself. It could be much easier to observe the technique from that [360° video.] (H5)*

However, the 3^rd^-person perspective also has its merits. Two participants wished to view the demonstration from another perspective in order to see the whole body posture. It was also suggested that the student could switch between these two viewpoints when necessary. In addition, one participant reflected on the possibility of moving in a virtual space and watching the demonstration from a desirable viewpoint.

#### 3.4.2 HMD and concentration

Half of the participants said that the HMD helped them to focus on the object of learning. For example, the limited field of view was one element that helped focusing. One a participant said:

*When you have the glasses [HMD] on, you must direct your attention exactly to it [lesson]. (H3)*

Concerning this matter, three participants mentioned that a traditional craft learning situation in a workshop may include many distractions: a student's attention may be distracted by another student's actions, or by noises or objects in the surrounding learning environment. However, HMD eliminated these distractions. For example, one student commented:

*It was nice that there were no noises, or anything redundant in the background. There was just the lesson. Therefore I could probably focus on that better. (H4)*

Another participant in turn considered that the novelty of the device may have led to better concentration. However, the opportunity to look around 360° made it hard to find the desirable object. Three participants said that they did not know where they should look from time to time, which distracted them from the topic of the lesson.

#### 3.4.3 Skill construction – interaction with materials, tools and interface

The majority of the participants (7/8) mentioned the importance of practicing by doing in craft learning. Working “hands on” was said to be the best way to learn hand craft skills. Half of them emphasized that in order to familiarize oneself with tools and materials, one must personally touch and feel them for real, i.e. interact; especially when the skill, material or tool is new.

*There are many people who don't use tools on a daily basis or have even seen one. Hence, they can't know its weight, how does it feel in the hand or what kind of effects it can have, wounds for example. And overall, how it (the tool) should be used, and the working postures. – It would be desirable to examine, that it [a knife] is actually sharp, because not everyone realizes that. (H1)*

Half of the participants wished that they could interact in the simulated environment. In this test the participants were only able to look around in the space. For example, one participant said that it would be nice if one could “click” the environment. Another participant stated that s/he felt like being in an “another reality” in a way, but as an observer rather than as an actor.

One participant also reflected that one could hold a tangible object during the video session; in this test that was not possible since a handheld device was used. Similarly, one participant also stated that muscle memory cannot be achieved via watching a video. However, another participant speculated that the authentic skill learning process might be forgotten if game controllers were used to simulate carving, instead of genuine tools.

In one interview the participant speculated that it is important to be able to train a skill in practice right after the lesson, when it is still “fresh in the memory.” For example, the time gap between the lessons and carving was approximately one week in this experience.

#### 3.4.4 Social interaction in skill learning

Half of the participants stated that a 360° VLE can be used to gain instructions and examples, although this depends on the skill: when the technique is new or considered difficult, the teacher's presence is crucial. For example, one participant said:

*The teacher should be there next to one, because when one begins to work, s/he can also help. Not just the video. – If you start working and you don't know what you are doing, the teacher is at least supporting.” (H6)*

This also implies that it is important to obtain feedback from a teacher in order to be able to proceed working in the right direction. Further, concerning support, three participants thought that 360° VLE lacks the ease of asking for help. For example, spontaneous asking is not as easy as in a traditional lesson. Although it is possible to ask questions after the class, the question may be forgotten during the video session. Hence, instant feedback was perceived to be important. On the other hand, questions asked during the video may cause disturbance if there are several students present. Moreover, one participant noted that the presence of the human (teacher) in a traditional lesson felt more natural compared to the video lesson, although s/he considered that this feeling might have arisen from his/her prejudices towards technology.

#### 3.4.5 Perceived learning outcomes

During the interview the participants were asked how the use of the 360° VLE impacted on their carving demonstration. Half of the participants reported that they tried to recall the video and intended to imitate the posture and carving technique they had seen. For instance, one participant said:

*It impacted instantly so that I took a similar posture and sat there in the same way. (H8)*

Moreover, another participant said that s/he would have wanted to take the indicated working posture while watching the video, but that the handheld device prevented that. Similarly, several participants noted that a headband would improve the viewing experience. Additionally, carving competence appeared to have an influence on participants’ perceptions of their learning. One experienced carver said that s/he did not learn anything new from the video about handling the knife. Similarly, another participant stated:

*I've carved as a child. Maybe it came more instinctively when I started to craft in that situation. Like how I have crafted before. (H6)*

## 4 Discussion

**Figure 3:**
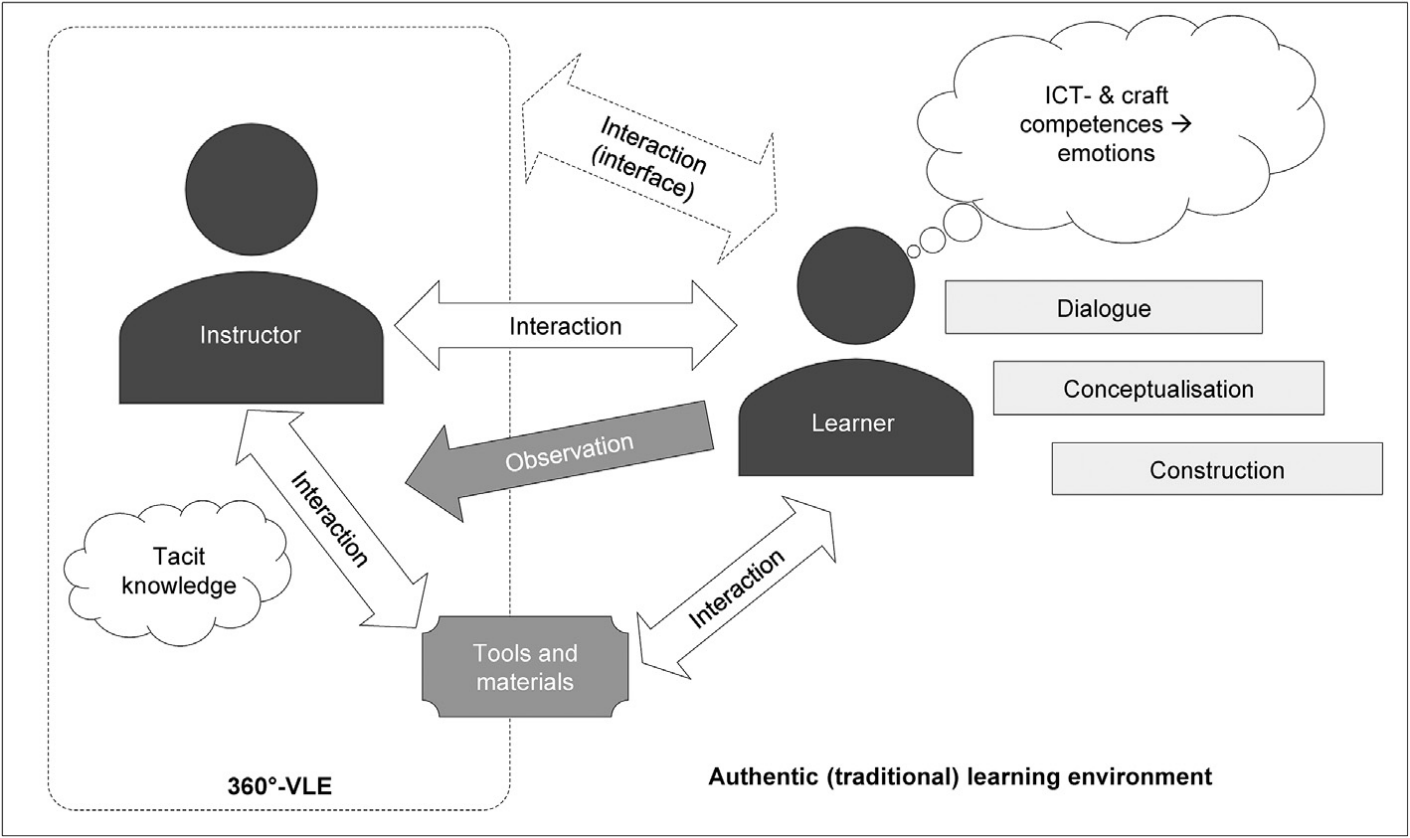
Interaction and craft learning processes in 360° virtual learning and traditional environments.

This study examined university students’ perceived satisfaction of a first-person perspective 360° VLE and a HMD in a handcraft skill-learning process. Concerning satisfaction, we examined attitudes towards ICT, ICT- and craft competences and emotional experiences after craft lessons. Moreover, the learning outcomes of a specific craft skill between the 360° VLE group and the traditional teacher-directed group were examined. Because earlier studies in this context are not available, this paper provides valuable insights into research fields concerning the pedagogy of the use of HMDs and 360° VLEs, craft and skill learning processes, learner satisfaction and the comparison of ICT-methods with traditional face-to-face learning. A summary of this pedagogical framework and of the findings of the study is presented in Figure 3.

### 4.1 Learning outcomes

It is particularly noteworthy that the traditional teaching method and the 360° VLE were found to be equally efficient according to the learning outcomes. Furthermore, no earlier studies comparing craft skill learning outcomes between traditional face-to-face lessons and 360° VLEs are available. However, other studies have indicated that equal learning outcomes can be achieved via e-learning and face-to-face courses (e.g. Dixson, 2010; Furió et al., 2015; Soffer and Nachmias, 2018), although the learning contents in these studies have usually included declarative knowledge rather than the procedural knowledge of which craft skills consist. The skill learning process also includes declarative and factual elements. Hence, this paper offers supplementary knowledge to the research field, in which various e-learning methods are compared to traditional courses.

It is important to pay attention to the specific affordances and boundaries of the 360° VLEs and HMDs, so that the use or rejection of the application is justified in a specific context, in this case skill and craft learning (see Bower, 2008). First of all, according to interviews of the experimental group, a common impression was that the 360° VLE and the HMD could be useful at least when learning “easy skills”, as carving was considered to be. In other words, the effectivity of 360° VLE depends on the learning content or on the quality and novelty of the skill. For example, ordinary video and interactive video can be equally effective media in learning basic knot tying (Nousiainen et al., 2008), whereas even very sophisticated VLE may be insufficient for skill learning if the skill requires multiple senses and fine motor control (Slater and Sanchez-Vives, 2016). Thus, the conclusion is that basic craft skills could be equally learned via a 360° VLE and a HMD.

### 4.2 Skill construction – interaction with materials, tools and interface

According to the interviews, interaction with a learning environment was found to be a core element of skill learning and satisfaction, which is well supported by earlier literature (e.g. Held and Hein, 1963; Zhang et al., 2006; So and Brush, 2008; Slater and Sanchez-Vives, 2016). In a craft learning process, the importance of genuine practicing in authentic environments and of concrete tools and materials was emphasized in the interviews. Similarly, Virtanen et al. (2017) found that medical students requested more hands-on training in the laboratory, although they were mainly satisfied with 360° VLE. Further, they noted that some students desired traditional lectures.

In this study, interactivity and immersion were created by the possibility for head movements, although more interaction with the 360° VLE was desired by some participants. This finding is understandable if compared to the study of Tham et al. (2018), in which a higher sense of agency in the virtual world was connected to motivation and engagement. Moreover, Dalgarno and Lee (2010) suggest that interaction in virtual learning environments (VLEs), including movement and object manipulation, provides potentially learning benefits. This is result of the spatial knowledge presentation that (a high-end 3D) -virtual learning environment affords.

However, genuine physical feedback, which is essential in skill construction, is highly challenging to accomplish via current technology (Jensen and Konradsen, 2018; Slater and Sanchez-Vives, 2016). Enhancing interactivity in the *observational features* of 360° VLE is possible to some extent, and was also suggested by the participants, i.e. enabling different angles of view (1^st^-person/3^rd^-person), moving in the virtual environment, clickable objects in the video and possibilities to return easily to displayed information.

Moreover, a HMD with a headband would be beneficial in observation, since it would free the hands and make mimicking possible while watching the video. Thus, it is suggested that 360° VLE is usable in skill observation, as long as the skill construction, i.e. physical practicing, takes place in authentic environments and with genuine tools. Furthermore, attention should be focused on the shift from observation to hands-on practice, since too long a time period between these phases may hinder recollection of the observed model.

### 4.3 Skill conceptualization – observational learning with 360° VLE

According to the interviews, 360° VLE with a 1^st^-person view appeared to clarify and concretize the working posture and tool handling. However, this did not apparently affect learning outcomes according to the Mann-Whitney U-test, since no statistically significant differences between the 360° VLE and traditional groups were observed in the working posture and technique. The experience of posture and tool handling becoming more concrete is however interesting, and understandable in the light of earlier literature concerning mental images and cognitive mapping (e.g. Slater and Sanchez-Vives, 2016). Watching actions from the third-person perspective was also requested as an option in addition to the 1^st^-person-perspective. This could indeed be a more informative perspective for certain skills, e.g. sawing, which is executed when standing.

According to the interviews, the lesson watched via HMD was considered to help focusing on the demonstration, whereas traditional demonstration in a classroom or workshop might include distracting features. This may be due to the nature of HMD, which eliminates environmental distractions (Higuera-Trujillo et al., 2017), and to the feeling of immersion, which fully engages the viewer (Rupp et al., 2019). However, visual and audio cues should be provided in order to guide the viewer's attention to the point where the demonstration takes place. Sarker (2017), for example, found that a lack of cues led to frustration, which was also observed in the current study.

Overall, the 1^st^-person perspective was considered practical particularly in the carving observation. Hence, it can be concluded that 360° VLE with a 1^st^-person perspective might be most valuable in the observation of fine motor skills, in which the core of the actions is placed in the hands and activities take place near the crafter's body. Such actions are also typically challenging for the instructor to demonstrate. For example, when there are many observers, the teacher is obliged to be placed further away in order for everyone to be able to see. Additionally, a traditional demonstration is generally seen as a “mirror image” even if the observer is close to the instructor, which can potentially complicate the learner's perception of the skill. Moreover, differences between handedness of the instructor and student may complicate the demonstration and observation situation. Thus, investigating affordances provided by 1^st^-person 360° VLE to solve these problems would be interesting. However, considering work safety in crafts, one should always be aware of the surroundings in a workshop. Thus, HMD should be used in a risk-free environment, as was done in this research.

### 4.4 Social interaction in skill learning and 360° VLE

In this study, the traditional method was seen as more suitable regarding the support provided by the teacher. Earlier studies have also shown that instant instructor feedback and timely response are associated with student satisfaction in e-learning (Sun et al., 2008). Particularly when the skill is trained in practice and craft competence is perceived to be low, the teacher's presence generates a feeling of safety. In e-learning courses, the importance of student-teacher interaction has been acknowledged, and opportunities for interaction needs to be created. However, due to procedural knowledge in skill learning, it is generally easier and more informative to provide practical answers to some questions. Anyhow 360° VLE is not a synonym for distance learning. Consequently, traditional and 360° VLE methods could be used concurrently, which was also concluded from the interviews. Nevertheless, it is necessary to consider what distance learning could mean with regard to interaction in a skill learning context.

However, properly scheduled 360° VLE usage could potentially enhance the quality of the teacher-student interaction, if communication with the instructor were to take place in the hands-on skill application phase in an authentic environment, after focused observation of the video, i.e. flipped learning (cf. Hirsto et al., 2019). Moreover, if the basis of a skill were observed beforehand via 360° video, the time in the classroom or workshop could be efficiently used for hands-on practice with teacher supervision. Hence, the instructor could concentrate on giving feedback, scaffolding and answering questions rather than on lecturing. It should be noted that the video demonstration should be of high quality with regard to e.g. postures, techniques and safety; moreover, teaching should always consider the learner's age, competence and motor development.

### 4.5 The relationship between ICT attitudes and ICT and craft competences and emotions

A surprising finding of this study was that no statistical significance regarding the relationship between ICT attitudes and positive or negative emotions after the craft class were observed. Consequently, a notable finding is that 360° and traditional lessons can be equally usable from the emotional point of view. This result has varied somewhat in earlier literature, in which negative ICT attitudes have led to negative emotions when using ICT (e.g. Saariluoma and Jokinen, 2014). However, in the experiment of Saariluoma and Jokinen (2014), participants also performed complex and novel tasks with an interface, leading to challenging situations and more intensive emotional reactions. In the current paper the only task was to view the 360° video. Nevertheless, this finding may indicate good emotional usability of the 360° VLE, since highly negative emotions were not observed in the 360° VLE group despite the negative attitudes. Moreover, the test situation may have included factors that have impacted positively on emotions, such as good usability and the presence of the instructor.

Regarding competences, the results indicated that students with stronger ICT competence experienced more positive emotions than those with lower competence. This finding is in line with existing literature (Juutinen & Saariluoma, 2010; Sun et al., 2008), in which high ICT competence is associated with positive emotions and satisfaction. Moreover, the results imply that a feeling of capability concerning crafts induces positive emotions, whereas a feeling of incapability of managing the subject matter leads to negative feelings. Hence, models concerning cycles of pride, competence and frustration in e-learning (Juutinen, 2011; Saariluoma and Jokinen, 2014) appear also to be applicable in a craft learning context. Ultimately, competence has a crucial role in the extent to which ICT is utilized in studies. For example, students with positive ICT experiences and high ICT competence may often use ICT voluntarily, leading to high attendance in ICT courses (cf. Wang and Newlin, 2002). Hence, student competences should be taken carefully into account, for example by providing assistance when planning courses that include specific skills, such as crafts and the use of (novel) technology.

### 4.6 Limitations

There are some limitations concerning the current study. First, because of the small sample and effect size, the results may only be applicable to the sample examined in this study. Moreover, since permission to interview impacted the group formation, it can be speculated whether unwillingness to participate in the interviews was due to negative attitudes toward ICT. Further, it should be noted that technically the learning outcome assessment measured the extent to which the participant carved according to the example shown in the 360° video or traditional lesson. Hence, the skill assessment excluded skillful performances that were not executed according to lessons. Moreover, although the control group and the experimental group were distributed according to craft competence, carving is quite a common craft skill in Finland. Thus, in future studies it could be interesting to test different skills with more diverse users and larger samples. Moreover, a pre-testing could be included in the experimental design. Finally, systematic qualitative examination of ICT attitudes and emotions should be considered.

## 5 Conclusions

Based on the results of the current study, we conclude that specific basic craft skills can be learned equally well via 360° VLE from the viewpoint of emotional usability and learning outcomes. It is suggested that 360° VLE is especially usable in skill observation, as long as the skill construction, i.e. physical practicing, takes place in authentic environments and with genuine tools. Moreover, HMD was considered to help focusing on the demonstration, whereas traditional demonstration in a classroom or workshop might include distracting features. However, careful examination of the learner's ICT and craft competences is recommended for the successful utilization of this technology, since competence appears to have a strong association with emotions. Moreover, opportunities for direct interaction with the instructor should be afforded both in craft learning and in ICT-mediated learning. Current global situation calls for extensive e-learning and distance learning solutions. This study suggests some key educational and pedagogical aspects that have to be considered when designing learning and teaching environments that include novel VLE-tools.

## Declarations

### Author contribution statement

Susanne Hallberg: Conceived and designed the experiments; Performed the experiments; Analyzed and interpreted the data; Contributed reagents, materials, analysis tools or data; Wrote the paper.

Laura Hirsto: Conceived and designed the experiments; Analyzed and interpreted the data; Contributed reagents, materials, analysis tools or data; Wrote the paper.

Jani Kaasinen: Conceived and designed the experiments; Contributed reagents, materials, analysis tools or data; Wrote the paper.

### Funding statement

This research was partially funded by Finnish Ministry of Education educational research and development projects DigiCampus (OKM/262/523/2017) and DigiPeda (OKM/199/523/2016, Docid: 311849).

### Competing interest statement

The authors declare no conflict of interest.

### Additional information

No additional information is available for this paper.
