# Supplementary material for: Experiences and outcomes of craft skill learning with a 360° virtual learning environment and a head-mounted display
Source: Heliyon. 2020 Aug 21;6(8):e04705. doi: 10.1016/j.heliyon.2020.e04705 (PMC7452434; doi:10.1016/j.heliyon.2020.e04705)
Supplement: Appendix 1 [file mmc1.docx]

**Appendix 1**. Interview themes and example questions

| Interview theme | Example questions |
| --- | --- |
| Free time ICT-use | - How do you utilize ICT in your free time? |
| Expectations about VR-360° -lesson | - What kind of expectations did you have about the VR-360°-lesson? |
| User experience of VR-360° -lesson | - How did the lesson meet your expectations? - What elements in the method did you like/dislike? - What would you change? |
| Broader context and possibilities for learning with VR-360° -lesson | - What would you think, if all the craft techniques in this course were instructed by this method? - In your opinion, what kind of influence the VR-360° method had on your learning? - If a new craft technique was instructed for you by this method, what would you desire? - What kind of VR360°-lesson would be ideal? |
